# Molecular Epidemiology of Laguna Negra Virus, Mato Grosso State, Brazil

**DOI:** 10.3201/eid1806.110948

**Published:** 2012-06

**Authors:** Elizabeth S. Travassos da Rosa, Daniele B.A. Medeiros, Márcio R.T. Nunes, Darlene B. Simith, Armando de S. Pereira, Mauro R. Elkhoury, Elizabeth Davi Santos, Marília Lavocat, Aparecido A. Marques, Alba V.G. Via, Vânia A. Kohl, Ana C.P. Terças, Paulo D`Andrea, Cibele R. Bonvícino, Elba R. Sampaio de Lemos, Pedro F.C. Vasconcelos

**Affiliations:** Evandro Chagas Institute, Ministry of Health, Ananindeua, Brazil (E.S. Travassos da Rosa, D.B. Medeiros, M.R. Nunes, D.B. Simith, A.S. Pereira, P.F. Vasconcelos);; National Foundation of Health, Brasília, Brazil (M.R. Elkhoury);; Surveillance Health Secretariat, Ministry of Health, Brasília (E.D. dos Santos, M. Lavocat);; Mato Grosso State Health Secretariat, Cuiabá, Brazil (A.A. Marques; A.V. Via);; Universitary Center Cândido Rondon, Cuiabá (V.A. Kohl);; Rural Producers Sindicate, Campo Novo do Parecis, Brazil (A.C. Terças);; Oswaldo Cruz Foundation, Rio de Janeiro, Brazil (P. D’andrea, C.R. Bonvicino);; National Câncer Institute, Rio de Janeiro (C.R. Bonvicino);; Pará State University, Belém, Brazil (P.F. Vasconcelos)

**Keywords:** Viruses, hantavirus, Laguna Negra, hantaviral pulmonary syndrome, respiratory infections, rodents, zoonoses, microenvironment, Brazil

## Abstract

We associated Laguna Negra virus with hantavirus pulmonary syndrome in Mato Grosso State, Brazil, and a previously unidentified potential host, the *Calomys callidus* rodent. Genetic testing revealed homologous sequencing in specimens from 20 humans and 8 mice. Further epidemiologic studies may lead to control of HPS in Mato Grosso State.

Hantavirus pulmonary syndrome (HPS) is a manifestation of an emerging zoonosis caused by New World viruses of the family *Bunyaviridae*, genus *Hantavirus*. Hantavirus is transmitted to humans by inhalation of aerosols of excreta from infected rodents of the subfamily *Sigmodontinae* (Rodentia, Cricetidae) (*1**,**2*). HPS was initially reported during an epidemic of severe respiratory disease that occurred in the southwestern United States in 1993 (*1*). HPS was subsequently identified in Brazil and other Latin American countries, which facilitated the recognition of new hantavirus species such as Laguna Negra virus (LNV), Andes virus, Choclo virus, Juquitiba virus, Araraquara virus, Castelo dos Sonhos virus, Anajatuba virus, as well as several other viruses detected in wild rodents which are not associated with HPS (*3**–**9*). Like particles of other bunyaviruses, hantavirus particles are spherical or pleomorphic and measure 80–120 nm in diameter; their genome comprises 3 RNA segments, and the small RNA fragment is used to characterize the nucleoprotein (N) gene and the hantavirus species (*2*).

During 1993–2009, a total of 1,246 cases of HPS were reported in Brazil; the state of Mato Grosso reported the fourth highest case count, diagnosed mainly in the municipalities of Tangará da Serra and Campo Novo do Parecis. However, the circulating hantavirus species and its host remained unknown, and identification of these factors were the main objectives of this study.

## The Study

Mato Grosso comprises 903,357.9 km^2^ and has an estimated population of 2,803,274 inhabitants living in 141 municipalities. Nineteen municipalities have reported cases of HPS, mainly near Brazil’s BR-364 highway, located between the north and southwestern sections of the state. The climate is equatorial subhumid, with an annual rainfall of 1,700 mm, and temperature range 24°–40°C; the landscape consists of savannah (*Cerrado*) and pre-Amazon rainforest. The economic activities are agricultural production and ecologic tourism.

**Figure 1:**
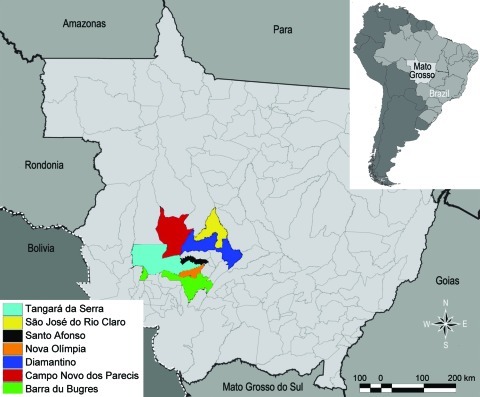
State of Mato Grosso, Brazil, indicating municipalities where hanta pulmonary syndrome cases occurred.

HPS was diagnosed in 24 persons who were IgM positive for LNV during 2001–2006 in the municipalities of Barra do Bugres (n = 1), Campo Novo do Parecis (n = 13), Diamantino (n = 3), Nova Olímpia (n = 1), Santo Afonso (n = 1), São José do Rio Claro (n = 1), and Tangará da Serra (n = 4) (Figure 1). Detailed information of patient samples submitted for nucleotide sequencing is provided in the Table.

During a 2001 ecological–epidemiologic study conducted in the municipalities of Tangará da Serra and Campo Novo do Parecis, researchers obtained blood and viscera samples from wild rodents (10). The researchers followed Brazilian Institute for the Environment and Renewable Natural Resources guidelines for the capture and handling of rodents and using biosafety level 3 protocols. The samples were tested for hantavirus; animals with positive test results were identified taxonomically by morphometry and molecular analysis of mitochondrial DNA (cytochrome b gene) (*10**,**11*).

For hantavirus detection, we conducted reverse transcription PCR to synthesize complementary DNA with generic hantavirus primers as described (*1**,**2*). We obtained N gene partial nucleotide sequences by using the Sanger method with the same primers (*3**,**4**,**6**,**9*). At least 3 amplicons per sample were sequenced in both directions to improve coverage and confidence for results. The obtained sequences were aligned with other hantavirus sequences available at the GenBank database (www.ncbi.nlm.nih.gov) with ClustalW software in BioEdit version 7.1.3 (www.mbio.ncsu.edu/BioEdit/bioedit.html). We implemented the maximum-likelihood and Bayesian methods by using PHYML (www.atgc-montpellier.fr/phyml/versions.php) and Mr. Bayes version 3.2 (http://mrbayes.scs.fsu.edu) software, respectively, for phylogenetic reconstructions. We used Modeltest version 3.7 (http://gel.ahabs.wisc.edu/mauve) to determine the best nucleotide substitution model. We analyzed 2 million replicates, with the sample fixed at every 1,000 trees generated, and used TRACER (www.evolve.zoo.ox.ac.uk) to determine whether the Bayesian analysis reached appropriate convergence (*3**,**6**,**9**,**12*).

We obtained amplicons from 20 of the 24 samples from persons with HPS and partial sequence of the N gene (≈434 bp) from 16 of the 24 samples from patients who were symptomatic at the time of sampling. During the ecologic study, 126 rodents were captured: 68 (53.9%) commensal synanthropic species, 49 (38.8%) wild rodents [*Calomys callidus* (n = 46), *Proechimys* sp. (n = 1), and *Necromys lasiurus* (n = 2)], and 9 (7.1%) unidentified species. IgG was detected in 8 (17.4%) *C. callidus* rodents (2 captured in Campo Novo do Parecis, 6 in Tangará da Serra). Amplicons were produced in lung/heart samples from 7 of the 8 IgG-positive rodents; 3 of those were selected for nucleotide sequencing of the N gene (Table).

**Figure 2:**
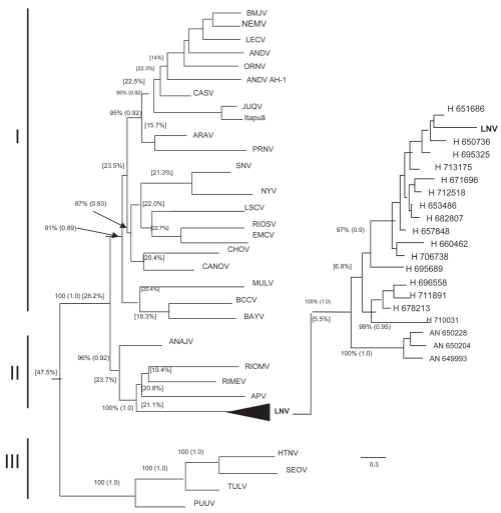
Phylogenetic comparison of the partial nucleotide sequences of nucleoprotein (N) gene of the small (S) RNA segment of different hantavirus strains from the Old World and New World by using the maximum-likelihood method and Bayesian analysis, with detail of the phylogenetic relationship between LNV strains isolated from humans and rodents in the state of Mato Grosso, Brazil. Bootstrap and Bayesian values (within parentheses) are shown for each respective knot. The arrows indicate the exact position of the values. Numbers within brackets correspond to the divergence between groups. Scale bar indicates 30% divergence of nucleotide sequences. APV, Alto Paraguay; ANAJV, Anajatuba, ANDV, Andes; BMJV, Andes Bermejo, NEMV, Andes Neembuco, LECV, Andes Lechiguanas; ORNV, Andes Oran; ARAV, Araraquara; BAYV, Bayou; BCCV, Black Creek Canal; CANOV, Cano Delgadito; CASV, Castelo dos Sonhos; CHOV, Choclo; EMCV, El Moro Canyon; HTNV, Hantaan; JUQV; Juquitiba-Araucaria; LNV, Laguna Negra; LSCV, Limestone Canyon; MULV, Muleshoe; NYV, New York; PRNV, Pergamino; PUUV, Puumala; RIOMV, Rio Mamoré; RIMEV, Rio Mearim; RIOSV, Rio Segundo; SEOV, Seoul; SNV, Sin Nombre; TULV, Tula.

All strains recovered from human and *C. callidus* rodent specimens were related and formed a monophylogenetic cluster with the LNV (GenBank accession no. AF005727), with a mean genetic divergence of 4.8%. These strains were included in subclade II, which comprises Anajatuba, Rio Mamore, Rio Mearim, and Alto Paraguay viruses (Figure 2). The genetic distance between strains recovered from rodents and humans was 5.5%, whereas the genetic distance between the human strains was 6.8%. Analysis of homology showed no difference between the partial amino acid sequences of human and rodent strains and LNV (100% homology). The homology of nucleotide sequences between the LNV strains was 89.9%–93.4% (Table A1). Most changes were silent mutations in the nucleotide sequences, indicated by the genetic divergence between LNV strains (Δdiv = 0.2%–9.8%).

LNV was initially confirmed in 1997 by serologic testing of a patient with HPS who died. The patient lived in Santiago, Chile, but was probably infected in Santa Cruz, Bolivia (*13*). In 1999, molecular analysis of the small N gene and medium Gn and Gc gene segments of the hantavirus genome in samples from HPS patients from Bolivia, western Paraguay, and Chile facilitated the genetic characterization of LNV and its association with the small vesper mouse *Calomys laucha*, which is considered the primary host of LNV. Subsequent studies in Argentina have also demonstrated the circulation of LNV in patients with HPS and in the large vesper mouse *Calomys callosus* (*4**,**9**,**13**–**15*).

## Conclusions

Our phylogenetic analysis of partial sequences of the N gene showed LNV as the cause of HPS, and the possible association of the organism with *C. callidus* rodents in western Brazil. These findings highlight the intense circulation of LNV in Matto Grosso municipalities located near the BR-364 highway. The vegetation and the equatorial climate of the area provide an excellent microenvironment for the maintenance of *C. callidus* rodents, as do areas in Bolivia Paraguay, and northern Argentina, where HPS caused by LNV has been reported (*4**,**9**,**13**–**15*).

The high nucleotide and amino acid homology between strains recovered from humans and the *C. callidus* rodent in Matto Grosso and the LNV prototype detected in Paraguay and Argentina suggest that LNV was transmitted by the rodent host *C. callidus* and led to the HPS cases that occurred in the vicinity of the highway BR-364 in southwestern Matto Grosso. No correlation was observed between the human LNV strains and year, geographic distribution, or between the severity of disease and the genetic diversity of LNV found in Brazil. The genetic data obtained in this study provide a better understanding of the molecular characterization of LNV and its association with HPS in southwestern Matto Grosso. Finally, on the basis of the phylogenetic analysis, the rodent species *C. callidus* is suggested as a potential reservoir for LNV. Further analyses of complete genome data are needed to confirm this result and to assess whether the *C. callidus* rodent is the sole carrier of of LNV in Matto Grosso.

## Appendix

**Table A1:** Homology between hantavirus strains originating from Mato Grosso, Brazil, and other hantaviruses from South America*

Strains	H 651686	H 650736	H 695325	H 713175	H 712518	H 671696	H 653486	H 682807	H 657848	H 660462	H 706738	H 695689	H 696558	H 711891	H 678213	H 710031	AN 649993	AN 650204	AN 650228	LNV	APV	RIMEV	RIOM	ANAJV	CASV	JUQV	ARAV
H 651686		98.1	96.8	95.1	95	94.1	95.6	95	95.7	93.5	94.6	92.9	91.6	91.6	92.1	90.1	92.4	93.4	92.8	96.8	71.1	72.7	76.9	72.7	75	72.7	77.2
H 650736	100		96.9	95.3	95.6	94.7	95.9	95.6	94.9	92.8	93.8	92.4	91.3	91.3	91.8	89.7	92.1	92.8	92.1	96.5	70.5	72.5	76.1	73.2	75.2	72.9	77.2
H 695325	100	100		97.1	95.2	94.6	96	95.7	95.9	93.8	94.9	92.7	91.6	91.6	92.1	90	94.3	95.2	94.4	94.1	71.3	71.8	76.3	72.5	74.8	73	76.7
H 713175	100	100	100		96.6	95.7	97.2	96.9	97.1	95.9	97.8	94.7	94.6	94.6	94	92.1	92.2	93.1	92.4	92.8	70.7	73.3	75.6	75	74.5	72.6	78.4
H 712518	100	100	100	100		98.5	99.1	98.5	98.7	96.8	97.8	96.3	95.3	95.3	95.7	93.7	91.8	91.8	91.8	93.8	70.8	73	76.3	77.4	75.5	73	78.2
H 671696	100	100	100	100	100		98.5	97.9	97.8	95.7	96.8	95.3	94.4	94.4	94.9	92.8	90.9	90.9	90.9	93.1	71.3	73.3	76.4	77.1	75.2	72.1	77.2
H 653486	100	100	100	100	100	100		99.4	99	96.9	97.9	96.3	95.3	95.3	95.7	93.7	91.8	91.8	91.9	93.5	71	73.5	76.4	76.9	75.5	73.3	77.2
H 682807	100	100	100	100	100	100	100		98.4	96.3	97.4	95.7	94.7	94.7	95.2	93.1	91.2	91.2	91.3	93.4	70.8	73.3	76	76.8	75.7	73	77.5
H 657848	100	100	100	100	100	100	100	100		97.8	98.8	96.6	95.6	95.6	96	94	91.9	92.2	92.1	92.8	70.7	73	75.9	77.1	75.2	72.6	77.7
H 660462	100	100	100	100	100	100	100	100	100		98.1	95.4	94.9	94.9	95.2	96.2	90.7	91	90.9	90.9	70.4	72.2	75.9	79.2	76	73.2	78.2
H 706738	100	100	100	100	100	100	100	100	100	100		96.6	96.8	96.8	96.2	94.3	91.9	92.2	92.1	91.9	70.5	72.7	76	77.2	75	72.6	77.5
H 695689	100	100	100	100	100	100	100	100	100	100	100		96.2	96.2	96.8	95.3	93.7	95.3	94.6	90.9	71.3	72.5	76.4	78	75	73.6	77.5
H 696558	100	100	100	100	100	100	100	100	100	100	100	100		100	99	96.8	94.4	92.5	92.5	90.2	71.1	73.5	76.7	78.3	76.2	73.2	77.7
H 711891	100	100	100	100	100	100	100	100	100	100	100	100	100		99	96.8	94.4	92.5	92.5	90.2	71.1	73.5	76.7	78.3	76.2	73.2	77.7
H 678213	100	100	100	100	100	100	100	100	100	100	100	100	100	100		96.6	94.6	93.1	93.1	90.6	71.1	73	76.6	78.1	75.5	73.3	77.5
H 710031	100	100	100	100	100	100	100	100	100	100	100	100	100	100	100		94.6	92.6	92.6	88.7	71.3	72.9	77	81	76.5	74.2	79.4
AN 649993	99.6	100	100	100	100	100	100	100	100	100	100	100	100	100	100	100		97.5	98.1	90.9	71.9	71.3	77.3	74.8	75.5	74.2	78.2
AN 650204	99.6	100	100	100	100	100	100	100	100	100	100	100	100	100	100	100	100		98.4	91.6	72.3	71.5	77.5	75	74.3	74.4	77.2
AN 650228	99.6	100	100	100	100	100	100	100	100	100	100	100	100	100	100	100	100	100		90.7	72.5	71.3	78.6	75	74	74.6	77
LNV	100	100	100	100	100	100	100	100	100	100	100	100	100	100	100	100	100	100	100		72.3	72.7	77.9	74.4	75.5	75.3	77.7
APV	96.7	96.7	96.7	96.7	96.7	96.7	96.7	96.7	96.7	96.7	96.7	96.7	96.7	96.7	96.7	96.7	96.3	96.3	96.3	96.7		76.6	76.6	79	76	72.6	76.2
RIMEV	94.8	94.8	94.8	94.8	94.8	94.8	94.8	94.8	94.8	94.8	94.8	94.8	94.8	94.8	94.8	94.8	94.2	94.2	94.2	94.8	96.2		76.8	78.9	72.8	70	72.8
RIOMV	97.7	97.7	97.7	97.7	97.7	97.7	97.7	97.7	97.7	97.7	97.7	97.7	97.7	97.7	97.7	97.7	97.1	97.1	97.1	97.7	99.1	97.3		81	74.3	73.2	79.9
ANAJV	96.2	96.2	96.2	96.2	96.2	96.2	96.2	96.2	96.2	96.2	96.2	96.2	96.2	96.2	96.2	96.2	96	96	96	96.2	96.7	95	93.8		76.2	75.7	77.2
CASV	91.5	91.5	91.5	91.5	91.5	91.5	91.5	91.5	91.5	91.5	91.5	91.5	91.5	91.5	91.5	91.5	91	91	91	91.5	89.7	56.6	56	57.5		79.4	77
JUQV	92	92	92	92	92	92	92	92	92	92	92	92	92	92	92	92	91.5	91.5	91.5	92	90.6	86.4	86.7	89.1	96.3		79.2
ARAV	90.6	90.6	90.6	90.6	90.6	90.6	90.6	90.6	90.6	90.6	90.6	90.6	90.6	90.6	90.6	90.6	90	90	90	90.6	90.6	56.1	56	57.5	95.6	93.9	

*Numbers above the diagonal indicate nucleotide sequence identity and numbers below the diagonal indicate amino acid sequence identity. Gray shading corresponds to the genetic relationship (nucleotides and amino acids) of hantavirus strains isolated from humans (H) and rodents (AN) with the Laguna Negra virus. APV, Alto Paraguay virus; ARAV, Araraquara virus; ANAJV, Anajatuba virus; CASV, Castelo dos Sonhos virus; JUQV, Juquitiba-Araucaria virus; LNV, Laguna Negra virus; RIOMV, Rio Mamore virus; RIMEV, Rio Mearim virus.

**Table:** Characteristics of human patients and *Calomys callidus* rodents with positive serologic results for hantavirus and partial nucleotide sequence of gene N, Mato Grosso, Brazil*

ID no.	Age, y/sex	Sample	Date of sample collection	Outcome	GenBank accession no.	Municipality
Humans						
H 678213	38/M	Serum	2005 Mar 10	Cure	JQ775513	Barra do Bugres (15°4′21′′S; 57°10′52′′W)
H 650736	33/M	Serum	2001 Dec 19	Death	JQ775504	Campo Novo do Parecis (13°40′31′′S; 57°53′31′′W)
H 660462	23/F	Serum	2002 Jul 8	Cure	JQ775506	
H 671696	20/F	Serum	2003 Aug 23	Death	JQ775505	
H 682807	20/M	Serum	2004 Aug 22	Death	JQ775507	
H 695689	27/F	Serum	2005 Aug 22	Cure	JQ775508	
H 696558	22/M	Blood	2005	Cure	JQ775512	
H 711891	42/M	Serum	2006 Aug 17	Death	JQ775503	
H 657848	42/M	Serum	2002 May 14	Cure	JQ775517	Diamantino (14°24′31′′S; 56°26′46′′W)
H 706738	13/F	Serum	2006 May 22	Cure	JQ775516	
H 712518	33/M	Serum	2006 Aug 29	Cure	JQ775518	
H 653486	13/F	Serum	2002 Feb 22	Cure	JQ775514	Nova Olímpia (14°47′50′′S; 57°17′17′′W)
H 695325	24/M	Serum	2005 Nov 11	Cure	JQ775515	Santo Afonso (14°29′51′′S; 7°0′7′′W)
H 713175	17/M	Serum	2006 Sep 26	Cure	JQ775509	São José do Rio Claro (13°26′48′′S; 56°43′17′′W)
H 651686	31/M	Serum	2002 Jan 22	Cure	JQ775511	Tangará da Serra (14°37′10′′S; 57°29′9′′W)
H 710031	7/F	Serum	2006 Jul 18	Death	JQ775510	
Rodents						
AN 650204	NA	Lung	NA	NA	JQ775500	Campo Novo do Parecis
AN 650228	NA	Lung	NA	NA	JQ775502	
AN 649993	NA	Heart	NA	NA	JQ775501	Tangará da Serra

*Source: Secretaria de Vigilância Epidemiológica do Estado do Mato Grosso and Instituto Evandro Chagas, Secretaria de Vigilância em Saúde), and Ministério da Saúde. NA, data not available.
